# Vasomotor fluctuations are increased in primary central nervous system lymphoma: a case–control study with fast functional MRI

**DOI:** 10.1093/braincomms/fcaf262

**Published:** 2025-07-08

**Authors:** Valter Poltojainen, Matti Järvelä, Janette Kemppainen, Nina Keinänen, Michaela Bode, Juha-Matti Isokangas, Hanne Kuitunen, Juha Nikkinen, Eila Sonkajärvi, Vesa Korhonen, Timo Tuovinen, Niko Huotari, Lauri Raitamaa, Janne Kananen, Heta Helakari, Tommi-Kalevi Korhonen, Sami Tetri, Outi Kuittinen, Vesa Kiviniemi

**Affiliations:** Oulu Functional Neuroimaging, University of Oulu/Oulu University Hospital, Oulu 90029, Finland; Research Unit of Health Sciences and Technology, University of Oulu, Oulu 90220, Finland; Department of Diagnostic Radiology, Medical Research Center (MRC), Oulu University Hospital, Oulu 90220, Finland; Oulu Functional Neuroimaging, University of Oulu/Oulu University Hospital, Oulu 90029, Finland; Research Unit of Health Sciences and Technology, University of Oulu, Oulu 90220, Finland; Department of Diagnostic Radiology, Medical Research Center (MRC), Oulu University Hospital, Oulu 90220, Finland; Oulu Functional Neuroimaging, University of Oulu/Oulu University Hospital, Oulu 90029, Finland; Cancer and Translational Medicine Research Unit, University of Oulu, Oulu 90220, Finland; Anesthesiology, Oulu University Hospital, Oulu 90220, Finland; Research Unit of Health Sciences and Technology, University of Oulu, Oulu 90220, Finland; Department of Diagnostic Radiology, Medical Research Center (MRC), Oulu University Hospital, Oulu 90220, Finland; Department of Diagnostic Radiology, Medical Research Center (MRC), Oulu University Hospital, Oulu 90220, Finland; Oncology and Hematology, Oulu University Hospital, Oulu 90220, Finland; Research Unit of Health Sciences and Technology, University of Oulu, Oulu 90220, Finland; Oncology and Radiotherapy, Oulu University Hospital, Oulu 90220, Finland; Anesthesiology, Oulu University Hospital, Oulu 90220, Finland; Oulu Functional Neuroimaging, University of Oulu/Oulu University Hospital, Oulu 90029, Finland; Research Unit of Health Sciences and Technology, University of Oulu, Oulu 90220, Finland; Department of Diagnostic Radiology, Medical Research Center (MRC), Oulu University Hospital, Oulu 90220, Finland; Oulu Functional Neuroimaging, University of Oulu/Oulu University Hospital, Oulu 90029, Finland; Research Unit of Health Sciences and Technology, University of Oulu, Oulu 90220, Finland; Department of Diagnostic Radiology, Medical Research Center (MRC), Oulu University Hospital, Oulu 90220, Finland; Neurosurgery, Clinical Neuroscience, University of Oulu/Oulu University Hospital, Oulu 90220, Finland; Oulu Functional Neuroimaging, University of Oulu/Oulu University Hospital, Oulu 90029, Finland; Research Unit of Health Sciences and Technology, University of Oulu, Oulu 90220, Finland; Department of Diagnostic Radiology, Medical Research Center (MRC), Oulu University Hospital, Oulu 90220, Finland; Oulu Functional Neuroimaging, University of Oulu/Oulu University Hospital, Oulu 90029, Finland; Research Unit of Health Sciences and Technology, University of Oulu, Oulu 90220, Finland; Department of Diagnostic Radiology, Medical Research Center (MRC), Oulu University Hospital, Oulu 90220, Finland; Oulu Functional Neuroimaging, University of Oulu/Oulu University Hospital, Oulu 90029, Finland; Research Unit of Health Sciences and Technology, University of Oulu, Oulu 90220, Finland; Department of Diagnostic Radiology, Medical Research Center (MRC), Oulu University Hospital, Oulu 90220, Finland; Oulu Functional Neuroimaging, University of Oulu/Oulu University Hospital, Oulu 90029, Finland; Research Unit of Health Sciences and Technology, University of Oulu, Oulu 90220, Finland; Department of Diagnostic Radiology, Medical Research Center (MRC), Oulu University Hospital, Oulu 90220, Finland; Neurosurgery, Clinical Neuroscience, University of Oulu/Oulu University Hospital, Oulu 90220, Finland; Neurosurgery, Clinical Neuroscience, University of Oulu/Oulu University Hospital, Oulu 90220, Finland; Oncology and Hematology, Oulu University Hospital, Oulu 90220, Finland; Cancer Center, Kuopio University Hospital, Kuopio 70210, Finland; Faculty of Health Medicine, Institute of Clinical Medicine, University of Eastern Finland, Kuopio 70211, Finland; Oulu Functional Neuroimaging, University of Oulu/Oulu University Hospital, Oulu 90029, Finland; Research Unit of Health Sciences and Technology, University of Oulu, Oulu 90220, Finland; Department of Diagnostic Radiology, Medical Research Center (MRC), Oulu University Hospital, Oulu 90220, Finland; Biocenter, University of Oulu, Oulu 90220, Finland

**Keywords:** lymphoma, malignancy, brain physiology, pulsation, fMRI

## Abstract

Primary CNS lymphoma is an aggressive brain tumour. An accumulation of malignant cells around cerebral blood vessels may potentially impair the convection of cerebrospinal fluid within perivascular spaces. Recent evidence links an increased variation of the blood oxygen level–dependent signal, a marker for haemodynamic changes, to risk of mortality in lymphoma. In this study, we aimed to characterize the physiological source(s) of increased blood oxygen level–dependent signal variation in lymphoma and characterize the link between altered physiological pulsations and mortality. Thirty lymphoma patients (median age 66 years; 9 females) and 40 healthy age-matched controls (median age 62 years; 29 females) were scanned using an ultrafast functional MRI sequence. We extracted physiological brain pulsation frequency bands from functional MRI data: full band (0.008–5 Hz), very low frequency (0.008–0.1 Hz), respiratory (0.1–0.5 Hz) and cardiac (0.7–2 Hz). We compared the respective pulsation amplitudes between groups using non-parametric covariate-adjusted permutation tests and studied the link between region-specific pulsation amplitudes and mortality in receiver-operating characteristic (ROC) and survival analyses. The lymphoma group showed higher amplitudes in all brain pulsation bands (*P* ≤ 0.05), with a global increase in the very-low-frequency band. Additionally, we detected increased fluctuation amplitudes in regions extending beyond the macroscopically visible tumour areas. The very-low-frequency and respiratory bands showed a link to mortality in the lymphoma patients, very-low-frequency band being independent of other predictive markers. Increased very-low-frequency amplitude, reflecting propagating vasomotor waves, was the main source for the increased blood oxygen level–dependent signal variation in lymphoma. The patients dying during follow-up showed higher very-low-frequency and respiratory amplitudes compared with the surviving patients, implicating them as a potential prognostic marker.

## Teaser

Poltojainen *et al*. use ultrafast MRI technology in primary CNS lymphoma to analyse brain pulsations driving glymphatic CSF convection. While tumour areas have increased cardiorespiratory pulsations, increased vasomotor waves extend beyond anatomical tumour changes in diagnostic MRI with a significant relation to mortality independently of other established prognostic markers.

## Graphical Abstract

**Graphical Abstract:**
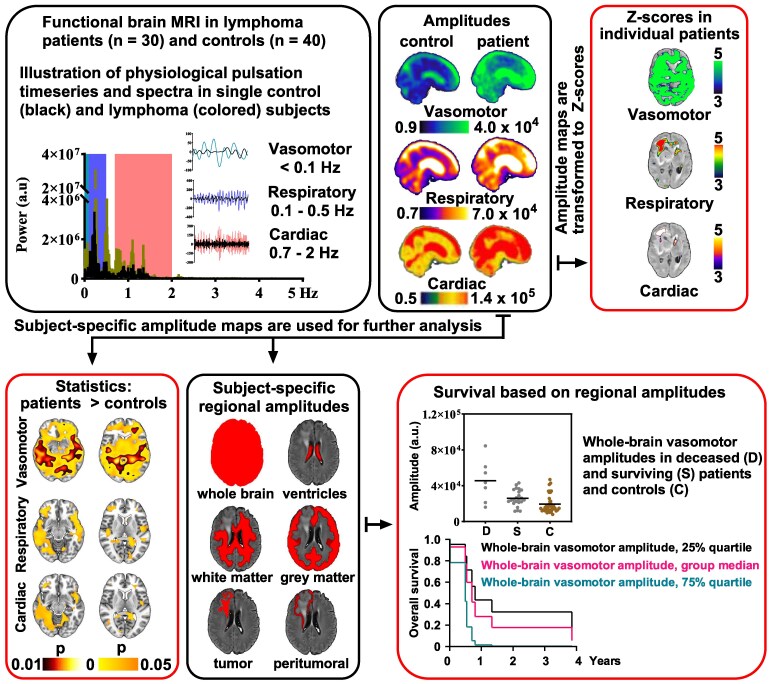

## Introduction

Primary CNS lymphomas (PCNSLs) are a rare type of non-Hodgkin lymphoma, with ∼1500 new cases annually in the USA^1^ and 50 cases in Finland representing one of the highest reported incidences in the world, with onset peaking among those aged 60–70 years.^2^ Mostly arising from lymphocytic B cells, PCNSLs have a diffuse pattern of whole-brain invasion that results in aggressive disease progression, with only 50% survival through the first year after diagnosis.^2^ Within a matter of weeks, patients may develop overt symptoms such as focal neurological deficits, behavioural changes and cognitive impairment. Headaches and vomiting may occur due to increased intracranial pressure. The perivascular PCNSL infiltration is abundantly evident to histological investigation^3-5^ and presents typical patterns in diagnostic MRI.^1,6^ However, structural neuroimaging methods may overlook sub-clinical foci, which could be seeding regions for more invasive disease progression.^5,6^

Traditionally, functional MRI (fMRI) has been applied to study very-low-frequency (VLF) blood oxygenation level–dependent (BOLD) signal that reflects spontaneous and task-dependent changes in brain activity.^7,8^ However, VLF activity can additionally reflect propagating (auto)regulatory vasomotor fluctuations^9-11^ and stationary wave responses to neuronal activation within cortical regions.^12-14^ The brain also shows high-frequency cardiorespiratory fluctuations in the fMRI signal.^10,15^ The mapping of the various physiological brain pulsations is drawing increasing attention for studying brain function in health and disease.^10,15-17^

The physiological pulsations arise from distinct mechanisms, but traditional fMRI techniques with slow sampling rates result in aliasing of the faster cardiorespiratory fMRI signal components over the 10-fold slower VLF fluctuations.^18^ However, recent technical innovations enable critically sampled fMRI-based techniques such as ultrafast magnetic resonance encephalography (MREG_BOLD_), which can separate cardiorespiratory signals from VLF fluctuations without aliasing.^18-20^

Evidence arising from ultrafast MREG_BOLD_ signals in studies of brain diseases implicate changes in specific physiological pulsations, such as increased cardiac pulsation in Alzheimer’s disease,^21,22^ increased respiratory pulsation in epilepsy^23^ and increased vasomotor VLF waves in narcolepsy.^24^ Importantly, in the absence of evident structural/anatomical changes to routine clinical neuroimaging, the MREG_BOLD_ technique can detect disease-related changes in seizure disorder patients^23^ and in PCNSL.^6^ In general, the findings of aberrant pulsations in specific brains regions are in accord with functional brain anatomy.^23^

Recent evidence shows that increased fMRI signal variation is a feature of PCNSL and that the magnitude of this physiological signal variation bears a relation to mortality at short-term follow-up.^6^ However, the exact source of physiological instability remained unresolved. Given the predominantly perivascular PCNSL invasion and subsequently altered brain (peri)vascular environment, we predict that PCNSL would increase the amplitude of MREG_BOLD_ vasomotor signals, which previous work has linked to vascular function.^11,25-27^

To examine the source of physiological fMRI signal variation in PCNSL, we deployed an ultrafast MREG_BOLD_ sequence in a series of patients and healthy controls and extracted the amplitude of fluctuation (AF) that correspond to established physiological brain pulsation mechanisms: full-band (AF_fB_), VLF (AF_VLF_), respiratory (AF_RESP_), and cardiac (AF_CARD_) bands. We thereby tested the hypothesis that pulsation amplitudes would be increased in PCNSL and used brain mapping to explore the spatial extent of the altered amplitudes. We found that the most significant and spatially broadest changes arose from increased vasomotor AF_VLF_ signals, which proved to be most pronounced in macroscopic tumour areas but extending to involve much of the cerebrum. Second, we found that AF_VLF_ and AF_RESP_ had a link with mortality in the short term, AF_VLF_ being independent of other predictive markers.

## Materials and methods

### Subjects

This study adheres to the Declaration of Helsinki and was conducted after obtaining institutional approval by the Ethical Committee of Northern Ostrobothnia Hospital District, Oulu University Hospital. Written informed consent was obtained from all subjects.

In this retrospective case–control study, our final analysis included 30 confirmed PCNSL patients (median age 66 years, 9 females) and 40 healthy age-matched controls (median age 62 years, 29 females).

The general inclusion criteria were absence of incidental findings in anatomical brain MRI [assessed by neuroradiologist (V.K.)], with acceptable fMRI data quality. We did not include outliers who have mean head motion larger than the isotropic 3 mm voxel size. There were some instances of non-neurological comorbidities among the patient and control groups. For complete demographics, see Table 1.

**Table 1:** Demographics

Parameter	Controls (*N* = 40)	PCNSL (*N* = 30)	Significance (*P-*value)
Age (years)	62 (61–66)	66 (60–71)	0.063 (ns)
Females (*n*)	29 (73%)	9 (30%)	0.0006 (*)
Relative motion (mm)	0.035 (0.029–0.044)	0.036 (0.032–0.044)	0.35 (ns)
Absolute motion (mm)	0.161 (0.113–0.219)	0.191 (0.149–0.323)	0.022 (*)
Framewise motion (mm)	0.061 (0.051–0.077)	0.067 (0.058–0.079)	0.21 (ns)
Respiratory rate (Hz)	0.25 (0.21–0.29)	0.25 (0.21–0.33)	0.51 (ns)
Cardiac rate (Hz)	1.12 (1.03–1.25)	1.19 (1.00–1.30)	0.71 (ns)
Comorbidity (*n*)			
Arrhythmia	1 (2.5%)	5 (17%)	
Asthma or sleep apnoea	4 (10%)	2 (7%)	
Coronary artery disease	0 (0%)	3 (10%)	
Hypercholesterolaemia	5 (13%)	5 (17%)	
Hypertension	7 (18%)	8 (27%)	
Hypothyroidism	2 (5%)	3 (10%)	
Psychiatric	0 (0%)	4 (13%)	
Deceased (*n*)	0 (0%)	7 (23%)	
Method for diagnosis (*n*)			
Resection		3 (10%)	
Needle biopsy		17 (56%)	
CSF aspiration		2 (7%)	
Unsolved due to referral		8 (27%)	
Diagnosis (*n*)			
DLBCL		29 (97%)	
Lymphocytic lymphoma		1 (3%)	
MREG scanning phase			
Before first treatment		22 (73%)	
Before second treatment		4 (13%)	
Before third treatment		3 (10%)	
Before fourth treatment		1 (3%)	
Seizures (*n*)		12 (40%)	

We present median values and corresponding interquartile range (IQR) limits, or number and corresponding %-portion. Statistical *P*-values represent results from an exact two-tailed Mann–Whitney U-test for continuous data or an exact two-tailed Fisher’s test for categorical data. The asterisk (*) denotes significant *P*-values; ns, non-significant difference. Note that some patients had been referred form other institutions after their pathologically confirmed diagnosis (stereotactic needle biopsy or resection), and due to restrictions regarding patient records, we could not ascertain the exact method for diagnosis in these cases.

DLBC, diffuse large B-cell lymphoma.

The preponderance of our present PCNSL cases (*n* = 21; 70%) and control subjects (*n* = 30; 75%) had been recruited for our previous study.^6^ For the present study, we had consecutively enrolled 45 cases suspected of PCNSL in initial radiological assessment or with certain diagnosis between April 2019 and September 2021 and assessed clinical outcome August 2024. After initial assessment for their eligibility and inclusion, we had retrospectively obtained a final diagnosis through stereotactic needle biopsy, tumour resection or CSF aspiration. Clinical evaluation also included excluding systemic lymphoma, excluding testicular lymphoma in male patients and evaluation for concomitant ocular involvement. We inspected the structural MR images visually and determined that the macroscopic effects of neurosurgical biopsy were minimal.^6^ We excluded 1 PCNSL patient due to excessive supra-voxel-scale head displacement and 14 suspected cases due to discordant histopathological diagnosis such as glioma.

Most PCNSL patients (*n* = 27; 90%) in the present study were receiving chemotherapeutic induction treatment with intra-arterial mannitol infusion to induce a transient blood–brain barrier disruption (BBBD) aiming to enhance intra-parenchymal concentrations of chemotherapeutics in pursuit of curative treatment.^28,29^ In this treatment, patients are first given a cycle of cytoreductive MATRix treatment consisting of cytarabine, methotrexate, rituximab and thiotepa. The actual BBBD-augmented chemotherapy regimen started some 4 weeks later and consisted of five drugs: rituximab, methotrexate, carboplatin, cyclophosphamide and etoposide. This BBBD-augmented treatment had been applied at 3–4-week cycles, with each cycle including 2 treatment days and two subsequent BBBD treatments. In brief, intravenous rituximab is given before each 2-day cycle, intravenous cyclophosphamide and etoposide are given shortly before each BBBD treatment and a combination of intra-arterial methotrexate and carboplatin is given after mannitol-induced BBBD via catheterization of carotid or vertebral arteries. Consolidation therapy consisted of high-dose carmustine–thiotepa combination chemotherapy and autologous stem cell transplantation.

We acquired MREG_BOLD_ imaging in PCNSL cases at the earliest possible treatment phase: before the first treatment in 22 patients (73%), before the second treatment in 4 (13%), before the third treatment in 3 (10%), and before the fourth treatment in 1 case (3%).

Seven of the 30 PCNSL patients (23%) died of their disease between April 2019 and August 2024. One patient had new diagnosis but was inoperable due to rapid disease progression. Four had received first-line BBBD treatment: only one had disease progression despite BBBD treatment, and they were offered palliative whole-brain radiation therapy (WBRT); one achieved curative response but some 2 years later had relapsed and died of rapid progression; one achieved curative response but died some 2 years later of unknown cause (we did not have access to complete patient records); and one achieved curative response but their fitness declined due to disease and related treatment, and recurrent infections, making them unrehabilitated. Two had relapsed PCNSL: one had reactions to MATRix treatment and was offered palliative WBRT and one had disease progression despite MATRix treatment and was offered palliative WBRT.

Furthermore, we consecutively enrolled 65 healthy control subjects and assessed their eligibility for inclusion between June 2018 and March 2021. We excluded two healthy control subjects due to incidental brain MRI findings and one potential control subject due to suspected early-stage Alzheimer’s disease. Given the typical PCNSL onset amongst the elderly, we accepted elderly control participants who were taking antihypertensive medication. We achieved age matching with 40 healthy control subjects.

### Scanning, image reconstruction and physiological monitoring

We performed fMRI using a Siemens Magnetom Skyra 3T MRI scanner (Siemens Healthineers AG, Munich, Germany) with a 32-channel head coil and an fMRI-based magnetic resonance encephalography (MREG_BOLD_) sequence: repetition time (TR) = 100 ms, echo time (TE) = 36 ms, flip angle (FA) = 25°, field-of-view (FOV) = 192 mm, 5-minute scan and isotropic voxel = 3 mm.^20,30,31^ Image reconstruction included a dynamic off-resonance in k-space method, which corrected for scanner warming and respiration-induced dynamic B_o_-field changes.^32^

We recorded heart rate using the in-scanner finger plethysmograph and respiration rate using the in-scanner respiration belt. Importantly, the critically sampled 10-Hz temporal resolution of the MREG_BOLD_ sequence enables precise separation of the physiological signal components without aliasing.^10,15,18^

During the same session, we obtained high-resolution T1-weighted 3D magnetization-prepared rapid acquisition with gradient echo images: TR = 1900 ms, TE = 2.49 ms, inversion time (TI) = 900ms, FA = 9°, FOV = 240 mm and isotropic voxel = 0.9 mm. In line with clinical routine, we also scanned the patients using a T2-weighted fluid-attenuated inversion recovery (FLAIR) sequence and a T1-weighted gadolinium-enhanced sequence to obtain a macroscopic estimation of tumour volumes. In seven patients, we had obtained the FLAIR datasets an average of three days before the MREG_BOLD_ acquisitions using standard imaging parameters on other scanners. Imaging parameters are shown previously.^6^

### Pre-processing

Pre-processing was identical to our previous study.^6^ We used FMRIB´s linear image registration tool to align the post-processed MREG_BOLD_ maps to the standard 3-mm Montreal Neurological Institute (MNI 152) brain template. To exclude non-brain voxels, we applied the resultant images to a binary brain mask including the ventricles. We performed further calculations using FSL 5.0.9, AFNI 20.1.2018, LIPSIA 3.1.0,^33^ Prism 9.1.0 and MATLAB software.

### Extracting physiological MREG_BOLD_ frequency ranges

To study the amplitudes of physiological brain pulsations from MREG_BOLD_ data, we formed periodogram data corresponding to the physiological pulsation mechanisms. First, we transformed the MREG_BOLD_ timeseries to the frequency domain using the *3dPeriodogram* function, which conducts a fast Fourier transformation (FFT). The frequency transformation of the MREG_BOLD_ timeseries yielded fB periodogram maps, wherein each voxel contains the fB spectrum (0.008–5 Hz). We then calculated average global MREG_BOLD_ frequency spectra from fB periodogram data with the *fslmeants* function.

Next, we determined subject-specific respiratory rates and cardiac rates for further pulsation analysis. To this end, we used finger plethysmograph frequency spectra, respiration belt frequency spectra and the global MREG_BOLD_ frequency spectra, which gives precise detection of physiological pulsation frequencies.^10,21-24^ Using these spectra, we determined cardiorespiratory rates automatically using the *findpeaks* function. Notably, since we determined cardiorespiratory rates separately from global MREG_BOLD_ frequency spectra and from physiological scanner data, we were able to verify subject-specific cardiorespiratory rates with great confidence.

**Figure 1:**
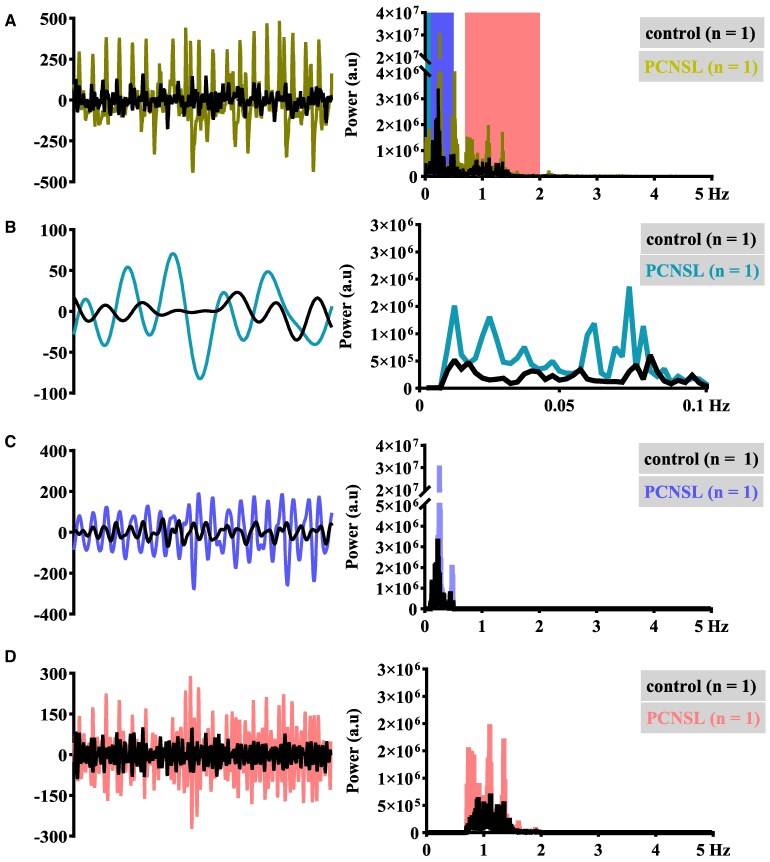
**Brain-wide physiological pulsation components are extractable from the ultrafast fMRI-based MREG_BOLD_ signal.** This figure illustrates in one representative PCNSL patient (*n* = 1; coloured lines) and in one control subject (*n* = 1; black lines) the brain-wide fB (0.008–5 Hz; **A**) MREG_BOLD_ timeseries (first 75 s of 5-min scan shown to highlight the waveform) and the corresponding frequency spectrum as obtained by FFT. In addition, the extracted physiological components of the MREG_BOLD_ signal and the corresponding frequency spectra are shown: VLF (0.008–0.1 Hz; **B**), respiratory (RESP; 0.1–0.5 Hz; **C**) and cardiac (CARD; 0.7–2 Hz; **D**). These physiological pulsation bands may be identified from the fB spectra. For the purposes of visualization, the *y*-axis is divided dived into two segments in the fB and RESP frequency spectra.

Finally, we applied the *fslroi* function on the initial fB MREG_BOLD_ periodogram data and formed additional periodogram data corresponding to the physiological brain pulsation bands: VLF (0.008–0.1 Hz), RESP (0.1–0.5 Hz; based on group minimum and group maximum respiratory rates) and CARD (0.7–2 Hz; based on group minimum and group maximum cardiac rates) (Fig. 1). Since we were also interested in how the band-passing method affects amplitude differences, we also formed alternative RESP_INDIVIDUAL_ (subject-specific respiratory rate ±0.05 Hz) and CARD_INDIVIDUAL_ (subject-specific cardiac rate ±0.05 Hz) periodogram data.

### Forming MREG_BOLD_ amplitude maps

To quantify the amplitudes of physiological pulsations, we first calculated amplitude maps from the MREG_BOLD_ VLF periodogram maps (AF_VLF_), following the amplitude of low-frequency fluctuations (ALFFs) method as developed previously.^16^ In addition to AF_VLF_, we calculated amplitudes from the non-aliased MREG_BOLD_ signal over the full-band (AF_fB_), respiratory (AF_RESP_) and cardiac (AF_CARD_) pulsation bands.^15^

To examine physiological pulsations in individual subjects, we additionally transformed the MREG_BOLD_ amplitude maps to respective *Z*-score maps, as described previously.^6^ We applied a *Z*-score threshold of 3.0, such that the final *Z*-score maps indicated voxels where the respective patient’s amplitude value exceeds the control population mean by at least three standard deviations.

To examine the group-level properties of these *Z*-score maps, we additionally formed cumulative incidence maps. To this end, we binarized the individual *Z*-score maps at a threshold 3.0 and then merged and summated the maps. In essence, these cumulative incidence maps depict the voxel-wise number of PCNSL patients who demonstrated markedly increased MREG_BOLD_ amplitudes according to our *Z*-scores ≥3 threshold. We formed a corresponding cumulative incidence map from the FLAIR imaging.

### Calculating pulsation amplitude within anatomical regions of interest

We calculated the individual mean MREG_BOLD_ amplitude values (excluding any null voxel values) from the following regions of interest (ROIs): whole brain, lateral ventricles, grey matter (GM) and white matter (WM) using standard 3 mm MNI 152 templates. We defined the individual macroscopic tumour areas as oedematous, hyper-intense areas in FLAIR imaging and segmented these areas manually as discussed previously.^6^ We next defined the adjacent peritumoural areas by dilating the initial binary tumour masks using the fslmaths *dilD* function. Importantly, to enable placing our focus on parenchymal pulsations, we excluded the lateral ventricles from the peritumoural ROIs and excluded macroscopic tumour ROIs from the GM and WM ROIs. Finally, we resampled all FLAIR images to 3 mm standard space. We used the final ROI-specific MREG_BOLD_ amplitude values in the subsequent survival analysis.

### Clinical factors in primary CNS lymphomas

The Memorial Sloan Kettering Cancer Center (MSKCC) score has some capability for PCNSL prognostics,^28,34^ whereby a higher MSKCC class predicts worse overall survival (OS): Class 1 (age <50 years), Class 2 (age ≥50 and Karnofsky performance score <70) and Class 3 (age ≥50 years and Karnofsky performance score <70). Other factors showing an association with worse OS in PCNSL include smaller number and greater volume of contrast-enhancing tumour areas in gadolinium-enhanced T1-weighted MRI.^33^ We included these clinical factors in our final analysis.

### Statistical analysis

We first examined the distributions of relevant parameters shown in Table 1 using normality testing within Prism and visual inspection. Next, we compared differences in age, mean absolute head displacement, mean relative head displacement, framewise displacement, respiratory rate and cardiac rate using exact two-tailed Mann–Whitney U-tests, also in Prism. We considered possible effects of head displacement, since head motion may degrade data quality and interfere with the separation of physiological brain pulsations.^35^ We compared differences in female portions between groups using an exact two-tailed Fisher’s test.

We examined differences in MREG_BOLD_ amplitudes at each voxel between the PCNSL and control subject groups using a threshold-free non-parametric two-sample *t*-test, using the *vlisa_2ndlevel* tool with 10 000 permutations and false discovery rate (FDR) correction.^36^ We corrected the permutation calculations for sex and mean absolute head motion, since only those parameters showed significant group differences. Additionally, we compared the respective mean, ROI-specific MREG_BOLD_ amplitudes between the PCNSL patients and control subjects using exact two-tailed Mann–Whitney U-tests in Prism. As control subjects naturally lacked tumour areas, we compared the amplitudes in macroscopic tumour areas and peritumoural areas in the PCNSL patients against their corresponding whole-brain amplitudes.

To examine if the region-specific MREG_BOLD_ amplitudes differentiated surviving PCNSL patients from deceased PCNSL patients, we conducted separate ROC analysis using the various ROI amplitudes. Additionally, we conducted an ROC analysis using the respective clinical factors: MSKCC score (score between 1 and3), age (years), *maximum* diameter of contrast-enhancing foci (mm), *total* sum of all contrast-enhancing tumour diameters (mm), number of contrast-enhancing foci and the volume of FLAIR hyper-intensity (mm^3^).

To formally establish a link between mortality and MREG_BOLD_ amplitudes, we conducted multivariate Cox regression analysis in Prism. To this end, we coded whole-brain AF_VLF_ amplitudes as categorical values (below or above the group median). Additionally, we included the following clinical factors within the mortality model: MSKCC score (score between 1 and 3), age (years), sex (female, male), *maximum* diameter of contrast-enhancing foci (millimetres) and number of contrast-enhancing foci.

To examine the correlations between mean ROI-specific MREG_BOLD_ amplitudes and clinical factors, we used non-parametric, paired Spearman coefficients. For all comparisons, we set the threshold for significance at *P* ≤ 0.05.

## Results

### Demographics

This retrospective case–control study included 30 (histo)pathologically diagnosed PCNSL patients and 40 healthy age-matched control subjects. Diagnosis of PCNSL had been confirmed by CSF aspiration in 2 (7%), stereotactic needle biopsy in 17 (56%), and resection in 3 (10%) patients. The precise method for biopsy (*stereotactic needle biopsy* versus *resection*) was unretrievable in eight (27%) cases who had been referred from other institutions after their histologically confirmed diagnosis, without conveyance of their complete electronic records.

Through our rigorous selection process, we age-matched the control subjects [median = 62 years; interquartile range (IQR) = 61–66 years] with the PCNSL patient group (median = 66 years; IQR = 60–71 years) (*P* = 0.063), corresponding with the most common age of PCNSL diagnosis in Finland.^2^ The portion of females was higher among control subjects (73%) than among the PCNSL patients (30%) (*P* = 0.0006), perhaps partly reflecting greater PCNSL incidence in males.^2^ While the magnitude of mean *absolute* head displacement was significantly lower amongst control subjects (median = 0.161 mm; IQR = 0.113–0.219 mm) than in the PCNSL patient group (median = 0.191 mm; IQR = 0.149–0.323 mm) (*P* = 0.022), the mean *relative* head displacement did not significantly differ between control subjects (median = 0.035 mm; IQR = 0.029–0.044 mm) and PCNSL patients (median = 0.036 mm; IQR = 0.032–0.044 mm) (*P* = 0.035). Notably, the magnitude of mean absolute and relative head displacement values remained low relative to the isotropic 3 mm voxel size. Similarly, there were no significant differences in framewise head displacement between control subjects (median = 0.061 mm; IQR = 0.051–0.077 mm) and PCNSL patients (median = 0.067 mm; IQR = 0.058–0.079 mm) (*P* = 0.21). There were no significant differences in respective respiratory rates between control subjects (median = 0.25 Hz; IQR 0.21–0.29 Hz) and PCNSL patients (median = 0.25 Hz; IQR = 0.21–0.33 Hz) (*P* = 0.51). Similarly, there were no differences in cardiac rates between control subjects (median = 1.12 Hz; IQR = 1.03–1.25 Hz) and PCNSL patients (median = 1.19 Hz; IQR = 1.00–1.30 Hz) (*P* = 0.71). The control and PCNSL subjects had identical minimum-to-maximum ranges for respiratory rates (0.11–0.41 Hz), whereas the minimum-to-maximum range of cardiac rates was higher in control subjects (0.91–1.92 Hz) than in the patients (0.78–1.38 Hz) (Table 1).

### Primary CNS lymphomas patients have brain-wide increases in physiological pulsation amplitudes

**Figure 2:**
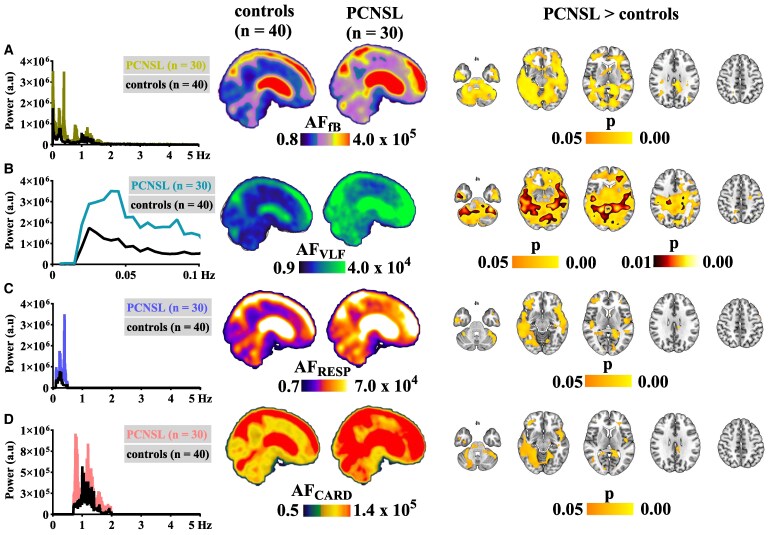
**Voxel-wise analysis shows that the amplitude of physiological pulsations is globally increased in brain of patients with PCNSL.** The MREG_BOLD_ sequence can capture normal and aberrant amplitudes of physiological pulsation over the entire brain: full-band (AF_fB_; 0.08–5 Hz; **A**), VLF (AF_VLF_; 0.008–0.1 Hz; **B**), respiratory (AF_RESP_; 0.1–0.5 Hz; **C**) and cardiac (AF_CARD_; 0.7–2 Hz; **D**). The left column illustrates the mean whole-brain MREG_BOLD_ frequency spectra in healthy age-matched controls (*n* = 40) and in PCNSL patients (*n* = 30). The sagittal brain slices represent corresponding mean amplitude maps from these groups. Rainbow colour bars represent average amplitude values; note that amplitude values between different rows are not directly comparable due to the different bandwidth lengths. The axial brain slices represent differences in the corresponding pulsation amplitudes (*P* ≤ 0.05 or *P* ≤ 0.01; non-parametric two-sample *t*-test, FDR corrected, corrected for mean absolute head displacement and sex). The anatomical background image is a standard T1-weighted MNI template.

We first visually inspected the amplitude-spectral properties of physiological fluctuations in the global MREG_BOLD_ frequency spectra. To this visual inspection, all frequency bands (fB, VLF, RESP and CARD) were of higher amplitude in the PCNSL group compared with controls. The most conspicuously higher amplitude was in the VLF frequency range (Fig. 2).

Voxel-wise statistical non-parametric analysis confirmed that the amplitudes of each physiological fluctuation were higher in the PCNSL group (*n* = 30) than in the age-matched control subjects (*n* = 40). As in the visual inspection of the mean spectra, the AF_VLF_ band showed the most statistically significant (*P* ≤ 0.01; non-parametric two-sample *t*-test, FDR corrected, corrected for sex and absolute head displacement) and spatially widespread increases, extending across the entire brain to encompass the following areas: cerebellum, pons, lateral ventricles, Sylvian fissures, frontal cortical areas, anterior temporal areas, occipital cortex and sub-cortical brain regions (caudate nuclei, accumbent nuclei, corpus callosum and hippocampi). These same areas also showed increased AF_fB_, AF_RESP_ and AF_CARD_ in the patient group (*P* ≤ 0.05; non-parametric two-sample *t*-test, FDR corrected, corrected for sex and absolute head displacement). However, the AF_fB,_ AF_RESP_ and AF_CARD_ increases were more circumscribed compared with the brain-wide AF_VLF_ increases. Importantly, the group differences in AF_fB_, AF_VLF_, AF_RESP_ and AF_CARD_ remained significant in the voxel-wise analysis even after excluding deceased PCNSL patients from the analysis (*P* ≤ 0.05; non-parametric two-sample *t*-test, FDR corrected, corrected for sex and absolute head displacement) (data not shown).

Voxel-wise analysis also showed that RESP_INDIVIDUAL_ (subject-specific respiratory rate ±0.05 Hz) amplitudes were increased in PCNSL as opposed to control subjects (*P* ≤ 0.05; non-parametric two-sample *t*-test, FDR corrected, corrected for sex and absolute head displacement), but the significant differences were more circumscribed than in the wider respiratory band. Interestingly, CARD_INDIVIDUAL_ (subject-specific cardiac rate ±0.05 Hz) amplitude maps did not show significant differences between groups (Supplementary Fig. 1). These findings persisted after excluding deceased PCNSL patients from the analysis (data not shown).

### Region of interest analysis shows that all pulsation amplitudes are increased in primary CNS lymphomas

**Figure 3:**
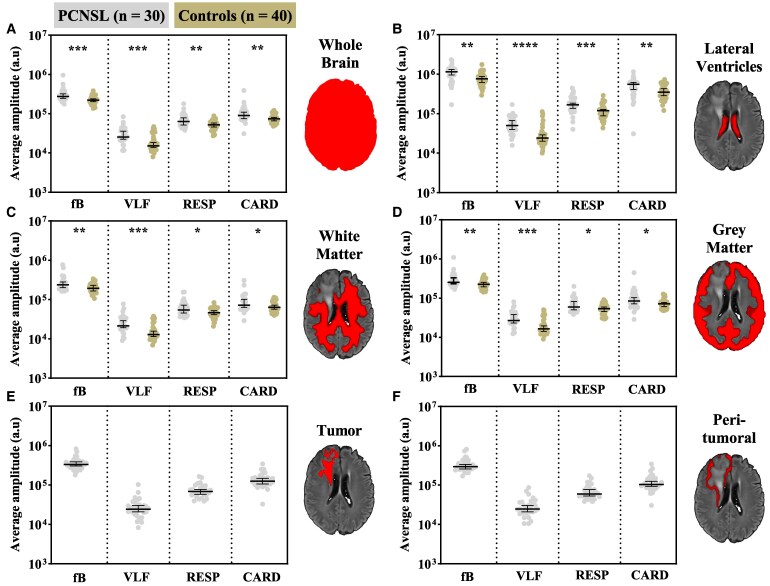
**ROI analysis shows brain-wide increases in the amplitude of physiological fluctuations in patients with PCNSL.** This figure illustrates average MREG_BOLD_ pulsation amplitudes in PCNSL patients (*n* = 30) and in age-matched control subjects (*n* = 40) in ROIs: whole brain (**A**), lateral ventricles (**B**), WM (**C**), GM (**D**), patient-specific macroscopically visible tumour area (**E**) and patient-specific peritumoural area (**F**). In PCNSL patients, we excluded the tumour areas from WM and GM areas, and CSF areas from the peritumoural areas. We defined the macroscopically visible tumour areas as hyper-intense FLAIR changes and peritumoural areas as ring-like areas surrounding the margins of the hyper-intense FLAIR changes. Horizontal lines represent group medians and corresponding 95% confidence intervals. The asterisk (*) denotes statistically significant group differences (*P* < 0.05) to exact two-tailed Mann–Whitney U-tests. We separated the following pulsation bands: full-band (fB; 0.008–5 Hz), VLF (0.008–0.1 Hz), respiratory (RESP; 0.1–0.5 Hz) and cardiac (CARD; 0.7–2 Hz. For tabular results, see Supplementary Table 1.

According to our ROI analysis, all pulsation amplitudes were significantly higher in the PCNSL group whole brain, lateral ventricle, WM and GM ROIs (Fig. 3; Supplementary Table 1). These findings were in line with the voxel-wise statistical analysis.

Technically, our voxel-wise statistical analysis cannot entirely account for individual disease-related alterations in vascular anatomy or differing tumour locations due to the averaging process. In this regard, the ROI analysis may complement the voxel-wise analysis. Relative to the mean whole-brain amplitudes in the patient group, the macroscopically visible tumour ROIs demonstrated significantly increased AF_fB_, AF_VLF_, and AF_CARD_. From these pulsation bands, only increases in AF_VLF_ diminished in peritumoural ROIs. AF_RESP_ alone was unaffected in both macroscopically visible tumour and peritumoural ROIs. Notably, the AF_CARD_ increase in tumour ROIs exceeded that of AF_VLF_ (Supplementary Table 1).

CARD_INDIVIDUAL_ amplitudes were unaffected, and RESP_INDIVIDUAL_ amplitudes were significantly increased in the PCNSL group whole-brain, lateral ventricle, WM and GM ROIs. These findings were again in good agreement with our findings from voxel-wise analysis. However, CARD_INDIVIDUAL_ amplitudes were significantly increased in both macroscopically visible tumour and peritumoural ROIs relative to the whole-brain ROIs in the patient group. RESP_INDIVIDUAL_ amplitudes were unaffected in both macroscopically visible and peritumoural ROIs (Supplementary Table 1).

### Very-low-frequency amplitude of fluctuation and respiratory amplitude of fluctuation are linked to survival

We next examined if ROI-specific MREG_BOLD_ amplitudes differentiated between the deceased (*n* = 7) from surviving PCNSL patients (*n* = 23).

**Figure 4:**
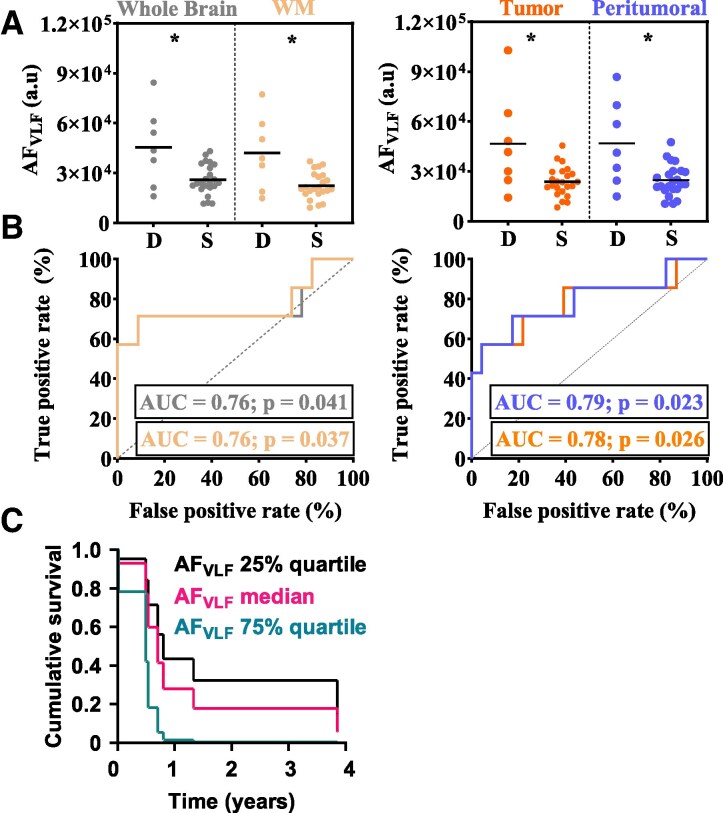
**The AF_VLF_ is linked to mortality in PCNSL.** (**A**) Average MREG_BOLD_ AF_VLF_ values in 7 deceased (D) and in 23 surviving (S) PCNSL patients, for the following ROIs: whole brain, WM, macroscopic tumour areas and peritumoural areas. Please note that GM and lateral ventricle AF_VLF_ values did not differentiate these groups and therefore are not illustrated here. The coloured circle symbols represent AF_VLF_ values in individual PCNSL patients, and horizontal black lines represent group median values. The asterisk (*) denotes statistically significant group differences (*P* < 0.05) to exact two-tailed Mann–Whitney U-tests. (**B**) Area under curve (AUC) generated by ROC analysis showing that region-specific AF_VLF_ values differentiated the deceased and surviving PCNSL patient subgroups. (**C**) Survival curves generated by a multivariate Cox regression model incorporating AF_VLF_, MSKCC score, age, sex, number of contrast-enhancing foci upon gadolinium-enhanced MRI and maximum diameter of all contrast-enhancing foci upon gadolinium-enhanced MRI in the PCNSL group (*n* = 30). This graph represents survival based on group median whole-brain AF_VLF_ and corresponding 25% and 75% quartiles.

In ROC analysis, we found that AF_VLF_ values differentiated the subgroups of PCNSL: whole brain [area under the curve (AUC) = 0.76; 95% confidence interval (CI) = 0.49–1.00; *P* = 0.041], WM (AUC = 0.76; 95% CI = 0.50–1.00; *P* = 0.037), tumour (AUC = 0.78; 95% CI = 0.55–1.00; *P* = 0.026) and peritumoural (AUC = 0.79; 95% CI = 0.56–1.00; *P* = 0.023) (Fig. 4). The lateral ventricle and GM AF_VLF_ were unable to differentiate patient subgroups (data not shown). Additionally, WM AF_RESP_ (AUC = 0.76; 95% CI = 0.56–0.96; *P* = 0.042), tumour AF_RESP_ (AUC = 0.76; 95% CI = 0.52–1.00; *P* = 0.037) and peritumoural AF_RESP_ (AUC = 0.76; 95% CI = 0.53–0.99; *P* = 0.042) differentiated these groups (data not shown).

The clinical factors considered in this study did not significantly differentiate deceased and surviving PCNSL patients: *maximum* diameter of contrast-enhancing foci, *total* sum of all contrast-enhancing tumour diameters, number of contrast-enhancing foci, MSKCC score, age and volume for macroscopically visible FLAIR hyper-intensity (data not shown).

According to our Cox regression analysis, whole-brain AF_VLF_ was linked to mortality. Indeed, based on a multivariate model, we calculated the following hazard ratios (HRs): above-median AF_VLF_ (HR = 4.21), increasing MSKCC score (HR = 2.43), increasing number of contrast-enhancing foci (HR = 1.70), increasing *maximum* diameter of contrast-enhancing foci (HR = 0.98), increasing age (HR = 0.89) and female sex (HR = 0.43). For visualization, we also formed additional survival curves showing whole-brain AF_VLF_ 25% quartile, median and 75% quartile curves (Fig. 4). In these, higher AF_VLF_ seems associated with worse OS, with the highest drop in OS during the first year.

### Very-low-frequency amplitude of fluctuation is independent of clinical factors

We calculated the Spearman correlation coefficients between ROI-specific MREG_BOLD_ amplitudes and the respective clinical parameters: *maximum* diameter of contrast-enhancing foci, *total* sum of all contrast-enhancing tumour diameters, number of contrast-enhancing foci, MSKCC score, age and the volume for FLAIR hyper-intensity.

None of the ROI-specific AF_VLF_ values correlated with these clinical parameters. However, lateral ventricle AF_fB_ significantly correlated with *total* sum of all contrast-enhancing tumour diameters (*r*_s_ = 0.37, *P* = 0.047) and with the number of contrast-enhancing foci (*r*_s_ = 0.48, *P* = 0.007). Tumour AF_fB_ significantly correlated with the number of contrast-enhancing foci (*r*_s_ = 0.43, *P* = 0.018) as did lateral ventricle AF_RESP_ (*r*_s_ = 0.41, *P* = 0.025). Whole-brain AF_CARD_ (*r*_s_ = 0.42, *P* = 0.021), WM AF_CARD_ (*r*_s_ = 0.39, *P* = 0.032), GM AF_CARD_ (*r*_s_ = 0.42, *P* = 0.023), lateral ventricle AF_CARD_ (*r*_s_ = 0.43, *P* = 0.017), tumour AF_CARD_ (*r*_s_ = 0.59, *P* = 0.001) and peritumoural AF_CARD_ (*r*_s_ = 0.39, *P* = 0.033) all correlated significantly with the number of contrast-enhancing foci. Additionally, the AF_CARD_ amplitude in tumour correlated with *total* sum of all contrast-enhancing tumour diameters (*r*_s_ = 0.39, *P* = 0.034). WM AF_CARD_ (*r*_s_ = 0.42, *P* = 0.022) and peritumoural AF_CARD_ (*r*_s_ = 0.37, *P* = 0.047) correlated positively with age (Supplementary Table 2).

### Subject-specific amplitude mapping reveals increased pulsation amplitudes within tumour areas, brain parenchyma and CSF spaces

Using the individual *Z*-score maps as described above, we compared within individual subjects the areas of altered pulsation amplitude with regions of FLAIR hyper-intensity. This analysis showed that there was considerable overlap between zones of increased AF_VLF_, AF_RESP_ and AF_CARD_ especially in tumour areas, with increased AF_VLF_ typically extending beyond the other changes to the WM, cortical GM and CSF areas (Supplementary Fig. 2).

These cumulative incidence maps show that increased MREG_BOLD_ amplitudes extended beyond the macroscopic tumour areas, as assessed by oedematous hyper-intense FLAIR areas. Indeed, the macroscopically visible tumour areas were typically located within the periventricular WM, whereas increased MREG_BOLD_ amplitudes were generally located more distally to the ventricles. The *Z*-score map of AF_VLF_ showed the greatest prevalence of increased AF_VLF_ within periventricular WM, cortical GM, the thalami and CSF areas with a mostly temporo-occipital distribution. The prevalence of increased AF_CARD_, and to a lesser extent increased AF_RESP_, were greatest within periventricular WM and cortical GM areas with a fronto-occipital distribution. Additionally, as opposed to AF_VLF_, the prevalence of increased AF_RESP_ and AF_CARD_ were also great within the caudate nuclei (Supplementary Fig. 3).

### Repeat scans show fluctuating MREG_BOLD_ amplitudes during treatment

We assessed the variability of MREG_BOLD_ amplitudes over time by repeat scanning between treatment rounds that had been applied at 3–4-week intervals. These acquisitions yielded repeat scans in 3 deceased patients and 14 surviving patients. Overall, we obtained five repeat scans in one patient, four scans in two patients each, three scans in four patients each and two scans in the remaining ten patients each. Considering all repeat scans, the median period between repeat scans was 28 days (IQR = 21–48 days).

In these data from repeat scanning, there was no compelling sign of systematically increasing or decreasing whole-brain pulsation amplitudes during treatment, but rather considerable individual fluctuation (Supplementary Fig. 4). This pattern of fluctuation was unaffected when we calculated the *maximum* whole-brain amplitudes or the total volume of all voxels exceeding the threshold *Z*-score ≥3.0 for a particular pulsation band.

### Increased MREG_BOLD_ amplitudes recur in the same tumour and CSF areas to repeat scanning

To assess the spatial areas that recurrently showed increased MREG_BOLD_ amplitudes during repeat scanning, we used a variation of the cumulative incidence maps described above (Supplementary Fig. 5). First, we calculated the *Z*-score maps by pulsation band from the repeat MREG_BOLD_ scans. We then binarized the individual maps at the threshold *Z*-score ≥3.0 and merged the repeat scans for each patient for summation to obtain the corresponding cumulative incidence maps. These summation maps revealed considerable persistence of the zones of increased AF_RESP_ and AF_CARD_ from scan to scan, mostly distinctly so within tumour and CSF areas. There was some persistence of zones with increased AF_VLF_ as well, but the volumes with increased AF_VLF_ were smaller than the corresponding volumes for AF_RESP_ and AF_CARD_.

## Discussion

This study aimed to detect the physiological sources of increased fMRI signal variability^6^ in patients with PCNSL. We deployed an ultrafast fMRI sequence (MREG_BOLD_) and calculated amplitude maps for groups of 30 PCNSL patients and 40 age-matched controls in pre-defined pulsation bands: full band (AF_fB_; 0.008–5 Hz), VLF (AF_VLF_; 0.008–0.1 Hz), respiratory (AF_RESP_; 0.1–0.5 Hz) and cardiac (AF_CARD_; 0.7–2 Hz). Taken together, all pulsation amplitudes were increased in the patient group, with the regions of elevated AF_VLF_ encompassing most of the brain and showing the most significant increases in periventricular WM. In our subject-level *Z*-score mapping, we found increased fluctuation amplitudes overlapping within macroscopically visible tumour areas, with increased AF_VLF_ extending beyond the cardiorespiratory changes to the WM, cortical GM and CSF areas. In our cumulative mapping, the macroscopically visible tumour areas typically located within periventricular WM areas, whereas increased fluctuation amplitudes extended from periventricular WM to the cortical GM and CSF areas, with increased AF_VLF_ additionally residing within the thalami and increased cardiorespiratory amplitudes within the caudate nuclei. In repeat MREG_BOLD_ scanning of 17 patients during treatment, whole-brain pulsation amplitudes showed considerable fluctuations over time, and spatially the increased cardiorespiratory amplitudes resided within tumour and CSF areas whereas the area of recurrently increased AF_VLF_ remained spatially smaller. Thus, our repeat scanning results suggest that, while AF_VLF_ remains elevated during patient follow-up, the localization of the increased AF_VLF_ changes with time. In survival analysis, elevated AF_VLF_ and AF_RESP_ were linked to mortality, AF_VLF_ being independent of other previously established clinical parameters.

### Primary CNS lymphomas increases vasomotor pulsations

There is general agreement that VLF fMRI signal fluctuations within the brain indirectly reflect neural activation. In brief, a localized neuronal activation may lead to arterial hyperaemia with a delay of some 3–5 s, which in turn increases the venous (over)oxygenation. In concert with these transient reductions in venous deoxyhaemoglobin concentrations, which acts as a paramagnetic contrast agent, the magnetic field distorts. This manifest as a transient, localized increase in the VLF signal, often lying to the implicated ensembles of activated neurons, which form functional networks via their axonal connections.^7,8^ This mechanism has been named the susceptibility-based BOLD effect. Meanwhile, resting-state fMRI studies have revealed larger scale propagating periodic VLF waves that move over the stationary boundaries of classical functionally connected brain regions.^12-14^ Some authors have additionally suggested vasomotor fluctuations contribute to the VLF signals, and by extension that these fluctuations vasomotor waves are involved in regulating metabolite homeostasis of brain tissue and intracranial pressure.^9-11,37^

In a healthy human brain, VLF signal fluctuations dominate within cortical GM areas.^15^ There are reports that AF_VLF_ increases in widespread brain regions during states of decreased arousal such as sleep,^11,25-27^ intravenous anaesthesia,^38^ midazolam sedation^39^ and narcolepsy.^24^

In the present study, we detected brain-wide increases in AF_VLF_ among PCNSL patients, most prominently within the periventricular WM and CSF areas. The most significant increases were located near vascular watershed areas, which are known as vulnerable border zones between vascular territories. In subject-level analysis, zones of markedly increased AF_VLF_ and other pulsation amplitudes extended beyond the margins of the macroscopically visible tumour areas. Among the three physiological pulsations, AF_VLF_ showed a link with short-term patient mortality, which proved to be independent of clinical factors such as MSKCC score and macroscopic tumour size estimates. Interestingly, we found that the increased AF_VLF_ fluctuated during treatment of surviving and deceased patients undergoing repeat scanning. Considering the inherent inter-scan variability of fMRI data, such trend should be further evaluated. However, the brain areas showing increased AF_VLF_ during follow-up remained relatively small and seemed to shift spatially between repeat MREG_BOLD_ scans.

Since the increased AF_VLF_ predominantly occurred within non-cortical areas, we are confident in attributing the VLF signals to vasomotor fluctuations rather than neural activations. Given the (peri)vascular nature of PCNSL invasion, and the spatial localization of our imaging findings, we suppose that increased AF_VLF_ mainly reflects an elevation of vasomotor activity in PCNSL. Indeed, there are several intriguing possibilities as to how PCNSL may affect the (peri)vascular space and vasomotor pulsation. Regional deterioration of the blood-brain barrier (BBB) in PCNSL^3,4^ could potentially impart a susceptibility to fluctuations in local cerebral vasomotion. Neuroinflammation and subsequent vasoactive signalling may be mediated by chemokines that have vasoactive properties and could possibly increase neurovascular reactivity in PCNSL patients.^40,41^ Among these, endothelin B, a vasoconstrictor, has also been implicated in immune escaping in PCNSL.^40^ The occlusion of the perivascular spaces that has been documented in PCNSL^3,4^ could also potentially affect perivascular cerebrospinal fluid flow dynamics. Alternative ways of vascularization have been characterized in PCNSL and have been linked to altered expression of cell surface molecules, such as aquaporin 4, that modulate the water flow and BBB stability.^42^ We suppose that these changes are likely to alter blood and CSF flow within the (peri)vascular environment. Since brain tumours tend to perturb the local metabolism and oxygenation status, this might additionally promote fluctuations in venous haemoglobin levels, CO_2_ and O_2_ levels. Furthermore, recent research has shown that awake mouse brain normally exhibits localized and transient spontaneous hypoxia pockets around veins.^43^ Given the avascular nature of PCNSL, the presence of hypoxic pockets could be an interesting subject for future studies.

### Primary CNS lymphomas increases respiratory pulsations

Respiratory fluctuations within the brain might arise from interactions between venous blood outflow and CSF inflow during the respiratory cycle.^44-46^ In brief, an inhalation lowers intrathoracic pressure, resulting in outflow of deoxygenated venous blood from the brain. The consequent decline in intracranial blood volume is compensated by inflowing CSF, as dictated by the Monro–Kellie doctrine that stipulates pressure equilibrium within the fixed cranial volume. Returning to the basis of BOLD signal changes, during inspiration, the deoxyhaemoglobin outflow leads to the transiently increased fMRI signal at respiratory frequencies.^10,15^ Alternatively, the respiratory signal fluctuations are related to flow-related enhancement effects, as previously magnetized spins enter the field of view.^17,44,45,47^ Respiratory spin-phase effects may also contribute to the respiratory signal; during inspiration, the water protons accelerate within the fixed magnetic field, such that their phase differs from that of water protons in stationary tissue. This phase difference may result in spin incoherence, magnetic field distortion and disrupted steady state free precession.^17,48,49^

In healthy human brain, respiratory signal fluctuations are the most widespread source of fMRI signal fluctuations. Respiratory signal fluctuations tend to predominate in (periventricular) WM areas^15^ and may be of increased amplitude in patients with epilepsy^23^ and during sleep.^25^ In the present study of PCNSL patients, we found increased AF_RESP_ in the same areas with the most significant elevations in AF_VLF_. Given the (peri)vascular changes in PCNSL, we suppose that the increased AF_RESP_ signals could potentially reflect altered venous blood flow within cortical areas and presumably altered perivenous CSF flow. We suppose that the venous oxygenation could additionally be involved in this process. Interestingly, as opposed to other pulsation mechanisms, there was no notable increase in respiratory pulsations within macroscopic tumour areas.

### Primary CNS lymphomas increases cardiac pulsations

Cardiac fluctuations within the brain likely arise from pressure waves driven by cardiac contractions that propagate along the arterial tree.^10,15,21,22,50-53^ In brief, propagating cardiac waves traverse from cerebral arteries into capillaries and perpendicularly into the peri-arterial space, finally proceeding into the interstitial space.^10,15,22^ Cardiac signal fluctuations arise from cardiac spin-phase effects, much as described above for respiratory waves. As such, the arrival of a systolic impulse in the brain evokes a momentary drop in fMRI signal, which is invariable located in (peri)arterial areas.^10,51^ Alternately, cardiac signals may arise from the local pulsation of the arteries themselves, resulting in spin density effects.^52,53^

In a healthy human brain, cardiac signal fluctuations seem to dominate near major arteries and in venous sinuses, but also occur within central CSF areas.^15^ The pulsation of arteries naturally decreases towards the distal vessels.^52^ A Doppler ultrasound study showed that increasing aortic stiffness leads to microvascular damage from excessive transmission of cardiac pulsatile energy towards the brain.^50^ Moreover, cardiac signal variability^21^ and propagation speed^22^ increases in Alzheimer’s disease, which is known to affect cerebral vasculature.

In the present study, the PCNSL patient group showed increased AF_CARD_ collocating with the most significant elevations in AF_VLF_ and AF_RESP_. Notably, when we focused only on the main cardiac peak itself, increased CARD_INDIVIDUAL_ amplitudes were not found in the voxel-wise statistical analysis. However, in the ROI analysis, we found increased CARD_INDIVIDUAL_ mainly confined within macroscopically visible (peri)tumoural areas. These results suggest that the amplitude of the main cardiac peak is itself increased within (peri)tumoural areas, but not in other brain ROIs of the PCNSL group. When extending the cardiac band, however, increased AF_CARD_ may be seen in multiple ROIs of the PCNSL brains. Therefore, additional physiological mechanisms may have contributed towards cardiac pulsations within the WM, GM and CSF ROIs that were not captured within the narrow cardiac peak. We are currently further evaluating the impact of these mechanisms.

### Mortality characteristics in primary CNS lymphomas

In general, the survival rate in PCNSL has long remained poor, with some 50% OS during the first year after diagnosis in Finland, after which the OS declines only modestly in time.^2^ In patients treated with BBBD-augmented chemotherapy, the 2-year OS is some 60%,^29^ and the median OS is 3.1 years.^28^ A subgroup analysis from a limited patient series showed that the patients treated with first-line BBBD therapy have a 100% 2-year OS, while in a relapsed setting the 2-year OS is reduced to 55%.^29^ In this study, 7 of the 30 PCNSL patients (23%) died. In brief, one patient was inoperable, two had relapsed PCNSL and were not given BBBD therapy and four had received first-line BBBD treatment.

Prognostic models have been developed in PCNSL as discussed previously.^6^ Greater MSKCC class predicts worse OS in BBBD-treated PCNSL patients, with the highest MSKCC risk class reflecting median OS 0.6 years.^28^ In the present study, we found a link between increased AF_VLF_ and AF_RESP_ and mortality in PCNSL, and our survival analysis showed that most increased AF_VLF_ values reflect OS less than a year. However, we also found that AF_VLF_ was independent of other clinical factors.

## Limitations

Among the limitations of this study, we note that our PCNSL patients and control subjects had some comorbidities (Table 1), which might have contributed to the observed effects. However, our results were robust to possible effects of group differences in age, sex, absolute and relative head displacement, framewise head displacement, respiratory and cardiac frequency and medication. Importantly, there were minimal structural or anatomical effects of the stereotactic needle biopsy procedure.^6^

While the study size was naturally constrained by recruitment within a reasonable time frame, we were able to recruit a relatively large sample of PCNSL patients despite disease rarity. Moreover, the high sampling rate of MREG_BOLD_ yielded a larger number of datapoints compared with conventional fMRI sequences. Due to the consequently unaliased fMRI signal acquisition and simultaneous physiological monitoring, we obtained good assignment of cardiorespiratory rates for their amplitude analysis.

The retrospective nature of this study calls for some caution in the interpretation of our discovery of a link between and AF_VLF_ and mortality. Indeed, while the MREG_BOLD_ is not yet a standard protocol, a status shared by other fMRI sequences, investigating this association in a prospective setting with sufficient sample size could better establish the MREG_BOLD_ procedure as a prognostic tool in PCNSL.

## Conclusion

Recent work has suggested that increased fMRI signal variation depicts haemodynamic changes that are linked to mortality in PCNSL. In this study, we show that vasomotor fluctuations are the most prominently increased brain pulsation band in PCNSL, with lesser increases in cardiorespiratory pulsations. Notably, increased vasomotor and respiratory amplitudes were associated with incipient PCNSL mortality, with vasomotor fluctuations being independent of other predictive markers. Moreover, we detected increased vasomotor fluctuations in brain areas distal to the macroscopic tumour margins. Based on previous fMRI studies and given the predominantly perivascular PCNSL invasion, we suggest that altered physiological fluctuations in PCNSL arise in relation to (peri)vascular disease features such as increased cerebrovascular reactivity, dysregulation of cerebral blood flow and increased perivascular cerebrospinal fluid pulsation. The present findings provide a novel pathophysiological understanding of PCNSL and present ultrafast fMRI as a potential prognostic marker for prognostic analysis and the assessment of treatment options.

## Supplementary Material

fcaf262_Supplementary_Data

## Data Availability

The data supporting this study’s findings are available from the corresponding author upon reasonable request.
