# Supplementary material for: Vasomotor fluctuations are increased in primary central nervous system lymphoma: a case–control study with fast functional MRI
Source: Brain Commun. 2025 Jul 8;7(4):fcaf262. doi: 10.1093/braincomms/fcaf262 (PMC12268500; doi:10.1093/braincomms/fcaf262)
Supplement: fcaf262_Supplementary_Data [file fcaf262_supplementary_data.docx]

**Vasomotor fluctuations are increased in primary central nervous system lymphoma: a case-control study with fast functional MRI**

Valter Poltojainen MD^1,2,3^, Matti Järvelä MD^1,2,3^, Janette Kemppainen MD^1,4^, Nina Keinänen MD^5^, Michaela Bode MD, PhD^2,3^, Juha-Matti Isokangas MD, PhD^3^, Hanne Kuitunen MD, PhD^6^, Juha Nikkinen MSc, PhD^2,7^, Eila Sonkajärvi MD, PhD^5^, Vesa Korhonen MSc, PhD^1,2,3^, Timo Tuovinen MD, PhD^1,2,3,8^, Niko Huotari MSc^1,2,3^, Lauri Raitamaa MSc^1,2,3^, Janne Kananen MD, MSc, PhD^1,2,3^, Heta Helakari MSc, PhD^1,2,3^, Tommi-Kalevi Korhonen MD, PhD^8^, Sami Tetri MD, PhD^8^, Outi Kuittinen MD, PhD^6,9,10^, Vesa Kiviniemi MD, PhD^1,2,3, 11^

**Affiliations**

1. Oulu Functional Neuroimaging, University of Oulu / Oulu University Hospital, Oulu, Finland
2. Research Unit of Health Sciences and Technology, University of Oulu, Oulu, Finland
3. Department of Diagnostic Radiology, Medical Research Center (MRC), Oulu University Hospital, Oulu, Finland
4. Cancer and Translational Medicine Research Unit, University of Oulu, Oulu, Finland
5. Anesthesiology, Oulu University Hospital, Oulu, Finland
6. Oncology and Hematology, Oulu University Hospital, Oulu, Finland
7. Oncology and Radiotherapy, Oulu University Hospital, Oulu, Finland
8. Neurosurgery, Clinical Neuroscience, University of Oulu/Oulu University Hospital, Oulu, Finland
9. Cancer Center, Kuopio University Hospital, Kuopio, Finland
10. Faculty of Health Medicine, Institute of Clinical Medicine, University of Eastern Finland, Kuopio, Finland
11. Biocenter, University of Oulu, Oulu, Finland

**Corresponding author**

Valter Poltojainen

E-mail: vpoltoja@univ.yo.oulu.fi

Address: Department of Diagnostic Radiology, P.O. Box 50, Kajaanintie 50, 90029, Oulu, Finland

**Supplementary Figures**

**
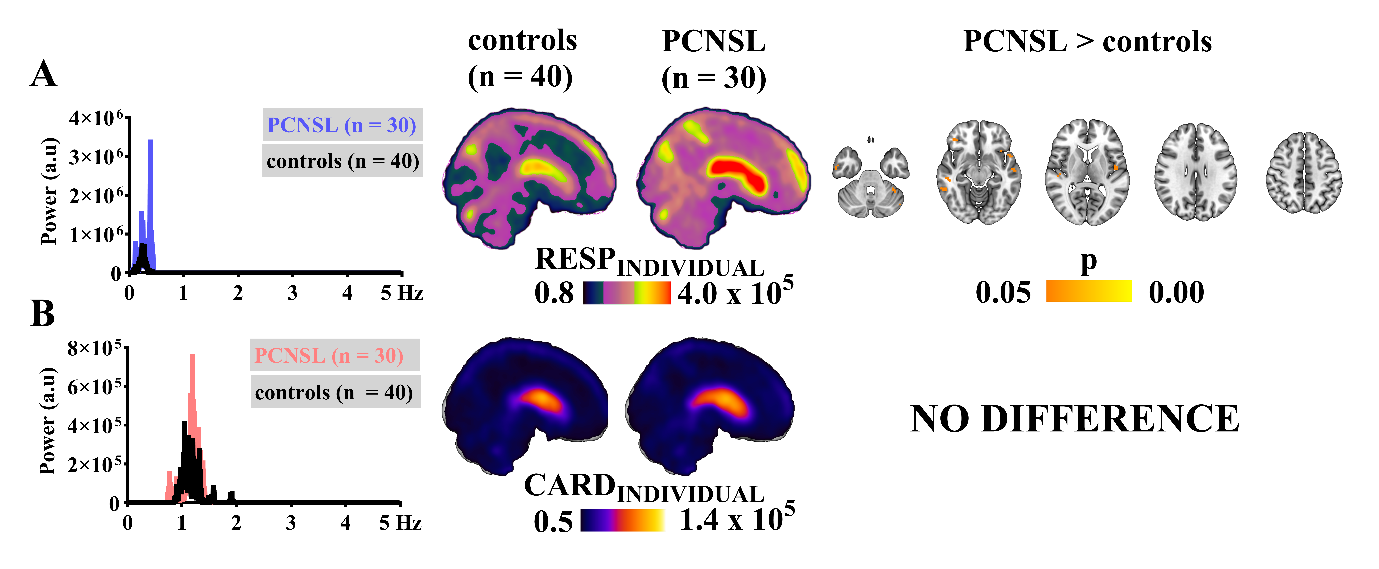
Supplementary Figure 1.** **An alternative band-passing method for analysis of Magnetic Resonance Encephalography (MREG_BOLD_) data in primary central nervous system lymphoma (PCNSL) (*n* = 30) and healthy control subjects (*n* = 40)**. **(A)** Individual band-passing method for MREG_BOLD_ respiratory pulsations (RESP_INDIVIDUAL_; subject-specific respiratory rate ± 0.05 Hz). **(B)** Individual band-passing method for MREG_BOLD_ cardiac pulsations (CARD_INDIVIDUAL_; subject-specific cardiac rate ± 0.05 Hz). The left column illustrates the mean whole-brain MREG_BOLD_ frequency spectra in PCNSL patients (*n* = 30) and in healthy age-matched controls (*n* = 40). The sagittal brain slices represent corresponding mean amplitude maps from these groups. Rainbow color bars represent average amplitude values; note that with this band-passing method, RESP_INDIVIDUAL_ and CARD_INDIVIDUAL_ amplitudes are comparable due to their similar bandwidth lengths. The axial brain slices represent differences in corresponding amplitudes (*p* ≤ 0.05; nonparametric two-sample t-test, False Discovery Rate corrected, corrected for mean absolute head displacement and sex). The anatomical background image is a standard T1-weighted Montreal Neurological Institute (MNI) template.

**
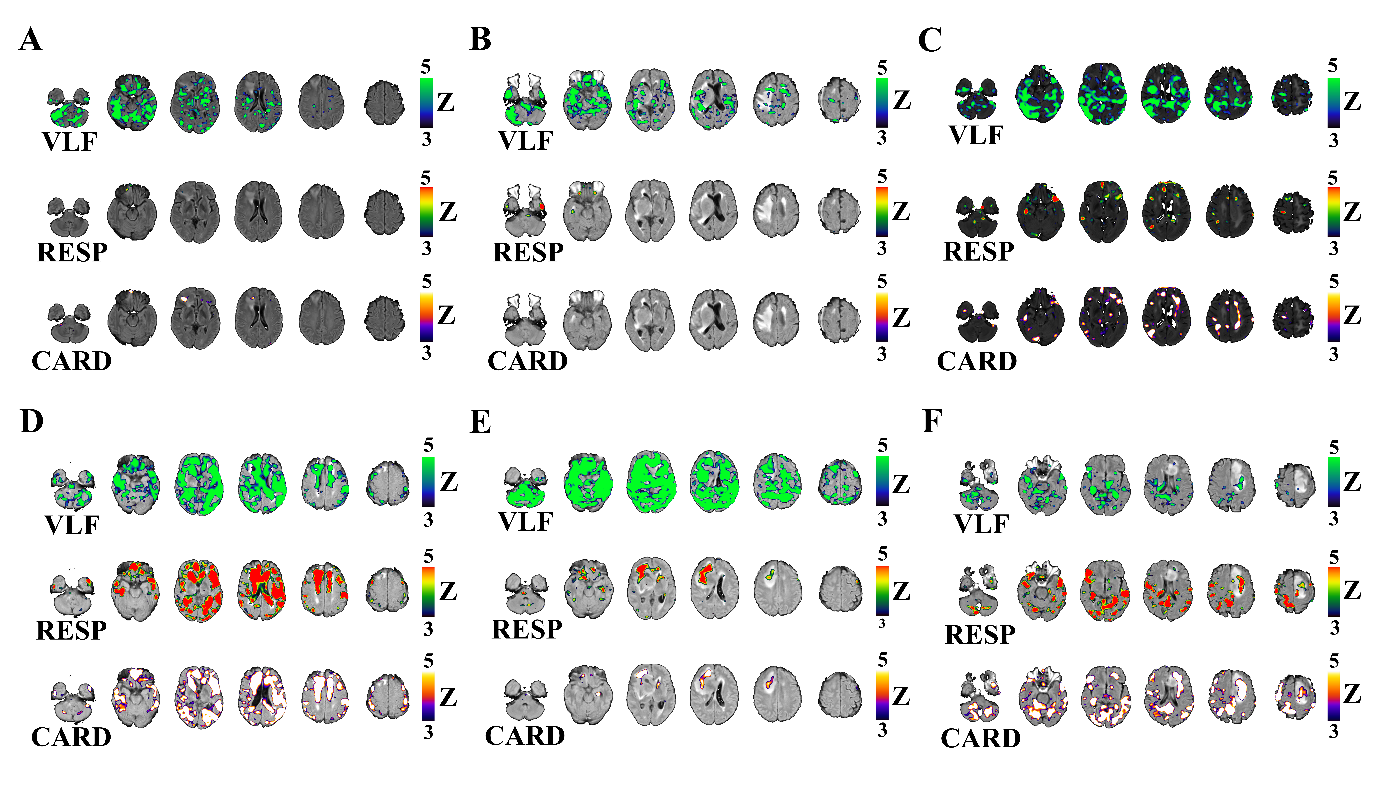
Supplementary Figure 2. Subject-level Z-score mapping shows increased Magnetic Resonance Encephalography (MREG_BOLD_) amplitudes within tumor areas, white matter, grey matter, and CSF spaces of primary central nervous system lymphoma (PCNSL) patients.** The figure depicts Z-score-encoded amplitude maps in a selected group of (*n* = 6; A to F) PCNSL patients. As per the definition of Z-scores, the colored clusters represent brain areas where the corresponding amplitude exceeded by at least three standard deviations (Z-score ≥ 3, color bar values) the mean in the control (*n* = 40) population. We present the following MREG_BOLD_ bands: very-low-frequency (VLF; 0.008 - 0.1 Hz), respiratory (RESP; 0.1 – 0.5 Hz), cardiac (CARD; 0.7 – 2Hz) in patients A to F. The background images are respective patient-specific anatomical T2-weighted Fluid-Attenuated Inversion Recovery (FLAIR) images whereby the macroscopically visible tumor areas are indicated by hyperintensity.

**
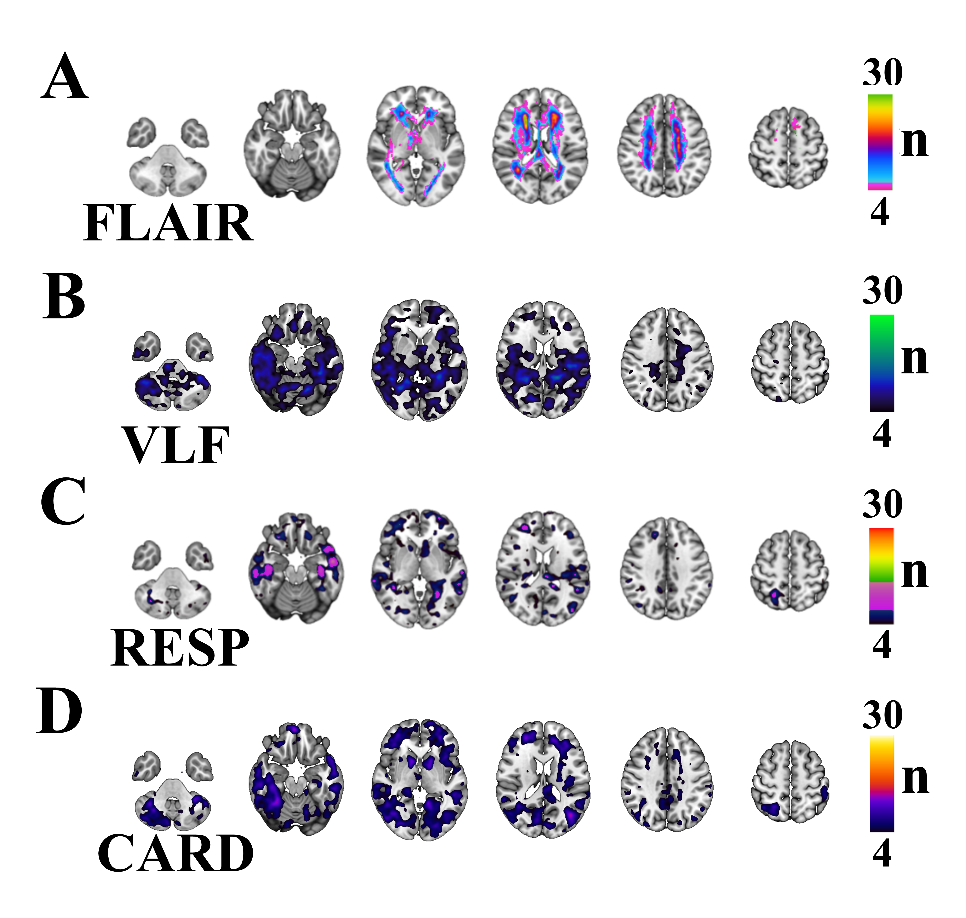
****Supplementary Figure 3. The cumulative incidence of findings from Magnetic Resonance Encephalography (MREG_BOLD_) and FLAIR imaging in the group of 30 primary central nervous system lymphoma (PCNSL) patients. (A)** The upper panel demonstrates the number of PCNSL patients (ranging from 4 to 30, see color bar values) who demonstrated hyperintensity in T2-weighted Fluid-Attenuated Inversion Recovery (FLAIR) imaging, reflecting macroscopically visible tumor areas. (**B to D)** The number of PCNSL patients who have “markedly increased MREG_BOLD_ amplitudes” or more specifically amplitude-derived Z-scores ≥ 3 compared to the control group, within the following MREG_BOLD_ bands: very-low-frequency (VLF; 0.008 - 0.1 Hz; **panel B**), respiratory (RESP; 0.1 – 0.5 Hz; **panel C**), cardiac (CARD; 0.7 – 2Hz; **panel D**). The anatomical background image is a standard T1-weighted Montreal Neurological Institute (MNI) template.

**
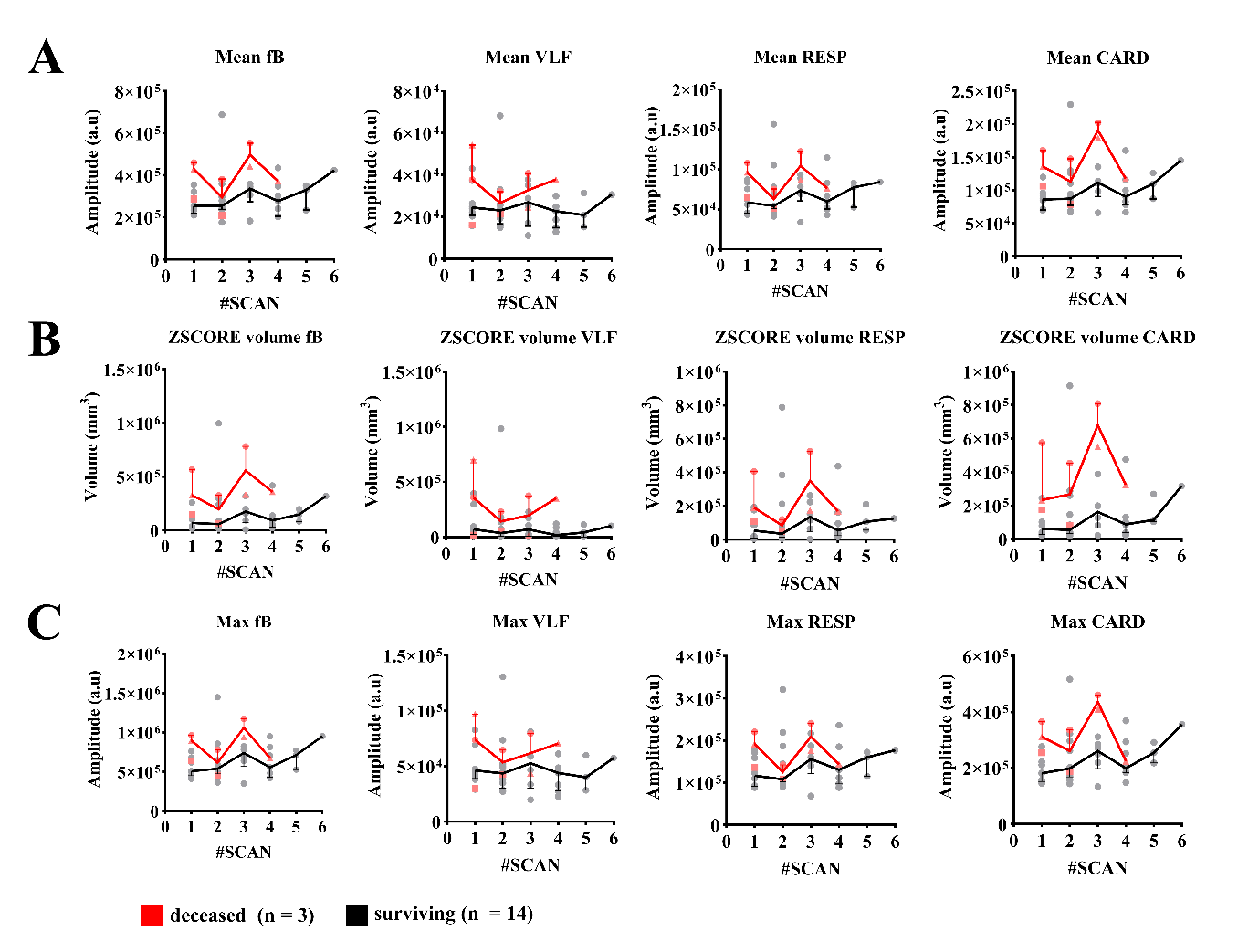
Supplementary Figure 4. Repeat Magnetic Resonance Encephalography (MREG_BOLD_) scans during treatment in surviving (*n* = 14, black color; individual subjects noted with grey circle symbols) and deceased (*n* = 3, red color; individual subjects noted light red triangle, square, and circle symbols) patients with primary central nervous system lymphoma (PCNSL)**. **(A to C)** The individual grey symbols represent values from individual surviving PCNSL patients (number of included subjects per scanning phase: 8, 12, 6, 7, 3, 1) and individual light red symbols represent values from individual deceased PCNSL patients (number of included subjects per scanning phase: 3, 2, 2, 1, 0, 0). Black lines in panels A to C represent respective group median values in surviving patients at given timepoints, and red lines represent respective group median values in deceased patients at given timepoints. The whiskers represent corresponding group interquartile ranges of those respective parameters. No statistical analysis has been used in this comparison. **(A)** The upper panel illustrates mean band-specific amplitudes within the whole-brain. **(B)** The middle panel represents volumes of the areas that have Z-scores greater than 3.0 within the whole brain. **(C)** The lower panel represents maximum band-specific amplitudes within the whole brain. The following pulsation bands are shown: full band (fB; 0.008 – 5 Hz), very-low frequency (VLF; 0.008 - 0.1 Hz), respiratory (RESP; 0.1 - 0.5 Hz), and cardiac (CARD; 0.7 - 2 Hz) bands. Note that not all 30 PCNSL patients yielded repeat scanning data.

**
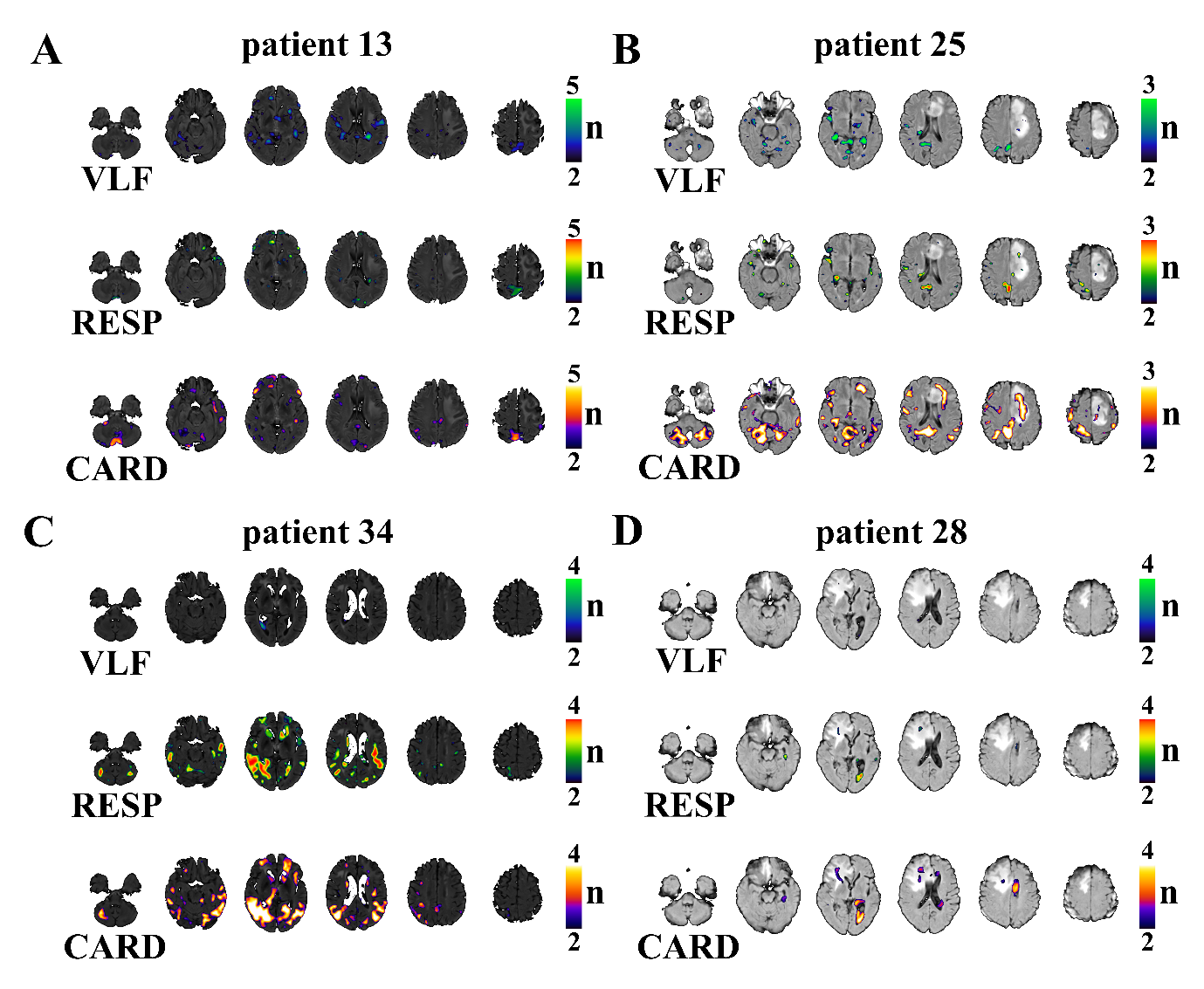
Supplementary Figure 5. Brain areas that *repeatedly* showed increased MREG_BOLD_ amplitudes to repeat scanning in primary central nervous system lymphoma (PCNSL) patients.** The figure illustrates cumulative incidence maps from a select set of patients (*n* = 4; panels A to D) with *repeated* MREG_BOLD_ scanning. The calculation is similar the that in the cumulative incidence maps shown in **Supplementary Figure 3** with the notable exception that these cumulative incidence maps are subject-specific rather than group-specific. In essence, these maps represent the number of repeated scans (2 to 5, see color bars) demonstrating “markedly increased MREG_BOLD_ amplitudes”, or more specifically the number of repeated scans that have Z-scores ≥ 3 compared to the control group within the following MREG_BOLD_ bands: very-low-frequency (VLF; 0.008 - 0.1 Hz), respiratory (RESP; 0.1 – 0.5 Hz), and cardiac (CARD; 0.7 – 2 Hz). The anatomical images on the background are patient-specific FLAIR images.

**Supplementary Tables**

**Supplementary Table 1.** Amplitudes of physiological fluctuations within anatomical regions of interest

| **Area and Frequency** | **Amplitude (x10^5^ a.u)** | | **Significance (*p*-value)** |
| --- | --- | --- | --- |
|  | **Controls (*n* = 40)** | **PCNSL (*n* = 30)** |  |
| **Whole brain** |  |  |  |
| Full Band | 2.21 [2.03 to 2.40] | 2.74 [2.46 to 3.23] | 0.0002 (***) |
| VLF | 0.15 [0.14 to 0.19] | 0.26 [0.22 to 0.36] | 0.0001 (***) |
| Respiratory | 0.52 [0.46 to 0.58] | 0.63 [0.51 to 0.78] | 0.0074 (**) |
| Cardiac | 0.74 [0.66 to 0.81] | 0.90 [0.75 to 1.10] | 0.0032 (**) |
| RESP_INDIVIDUAL_ | 0.21 [0.20 to 0.24] | 0.25 [0.21 to 0.30] | 0.014 (*) |
| CARD_INDIVIDUAL_ | 0.14 [0.12 to 0.15] | 0.14 [0.12 to 0.17] | 0.37 (ns) |
| **Lateral Ventricle** |  |  |  |
| Full Band | 7.60 [6.16 to 8.76] | 11.5 [9.54 to 13.2] | 0.0012 (**) |
| VLF | 0.24 [0.20 to 0.29] | 0.50 [0.40 to 0.67] | < 0.0001 (****) |
| Respiratory | 1.19 [0.88 to 1.28] | 1.68 [1.36 to 1.86] | 0.0008 (***) |
| Cardiac | 3.50 [2.85 to 4.32] | 5.54 [4.10 to 6.16] | 0.0051 (**) |
| RESP_INDIVIDUAL_ | 0.43 [0.36 to 0.56] | 0.65 [0.51 to 0.82] | 0.0037 (**) |
| CARD_INDIVIDUAL_ | 0.82 [0.59 to 1.10] | 1.00 [0.63 to 1.21] | 0.57 (ns) |
| **White Matter** |  |  |  |
| Full Band | 1.94 [1.65 to 2.29] | 2.34 [2.02 to 2.80] | 0.0019 (**) |
| VLF | 0.13 [0.12 to 0.16] | 0.21 [0.19 to 0.29] | < 0.0001 (****) |
| Respiratory | 0.46 [0.41 to 0.52] | 0.54 [0.45 to 0.72] | 0.016 (*) |
| Cardiac | 0.63 [0.57 to 0.72] | 0.72 [0.63 to 1.02] | 0.032 (*) |
| RESP_INDIVIDUAL_ | 0.19 [0.18 to 0.21] | 0.22 [0.19 to 0.27] | 0.035 (*) |
| CARD_INDIVIDUAL_ | 0.13 [0.11 to 0.15] | 0.14 [0.11 to 0.17] | 0.86 (ns) |
| **Grey Matter** |  |  |  |
| Full Band | 2.27 [1.95 to 2.53] | 2.55 [2.33 to 3.33] | 0.0051 (**) |
| VLF | 0.17 [0.14 to 0.20] | 0.27 [0.23 to 0.38] | 0.0004 (***) |
| Respiratory | 0.54 [0.46 to 0.60] | 0.60 [0.50 to 0.82] | 0.084 (*) |
| Cardiac | 0.71 [0.61 to 0.78] | 0.85 [0.72 to 1.03] | 0.035 (*) |
| RESP_INDIVIDUAL_ | 0.22 [0.20 to 0.24] | 0.24 [0.20 to 0.33] | 0.046 (*) |
| CARD_INDIVIDUAL_ | 0.11 [0.10 to 0.13] | 0.11 [0.10 to 0.15] | 0.75 (ns) |
| **Tumor** |  |  |  |
| Full Band |  | 3.33 [3.05 to 3.89] | Δ 0.0002 (***) |
| VLF |  | 0.24 [0.21 to 0.30] | Δ 0.014 (*) |
| Respiratory |  | 0.68 [0.58 to 0.77] | Δ 0.20 (ns) |
| Cardiac |  | 1.25 [1.06 to 1.47] | Δ < 0.0001 (****) |
| RESP_INDIVIDUAL_ |  | 0.28 [0.24 to 0.32] | Δ 0.084 (ns) |
| CARD_INDIVIDUAL_ |  | 0.24 [0.20 to 0.30] | Δ < 0.0001 (****) |
| **Peritumoral Area** |  |  |  |
| Full Band |  | 2.97 [2.58 to 3.34] | Δ 0.022 (*) |
| VLF |  | 0.25 [0.21 to 0.30] | Δ 0.077 (ns) |
| Respiratory |  | 0.60 [0.53 to 0.78] | Δ 0.86 (ns) |
| Cardiac |  | 1.03 [0.94 to 1.25] | Δ 0.0002 (***) |
| RESP_INDIVIDUAL_ |  | 0.26 [0.23 to 0.31] | Δ 0.24 (ns) |
| CARD_INDIVIDUAL_ |  | 0.20 [0.17 to 0.24] | Δ < 0.0001 (****) |

Note: The physiological pulsation bands as detected with MREG_BOLD_ were full band (fB; 0.008–5 Hz), very-low-frequency (VLF; 0.008 – 0.1 Hz), respiratory (RESP; 0.1–0.5 Hz), and cardiac (CARD; 0.7–2 Hz). The amplitudes from alternative band-passing methods are also shown: RESP_INDIVIDUAL_ (subject-specific respiratory rate ± 0.05 Hz) and CARD_INDIVIDUAL_ (subject-specific cardiac rate ± 0.05 Hz), which are directly comparable to AF_VLF_ since the bandwidths lengths are the same. In PCNSL patients, we excluded the macroscopically visible tumor areas (as defined as FLAIR hyperintensity) from white matter (WM) and grey matter (GM) areas, and CSF areas from peritumoral areas. These tabular values represent group median and corresponding interquartile range (IQR) limits. The *p*-values represent statistical results from exact two-tailed Mann-Whitney U-tests or (Δ) exact two-tailed Wilcoxon matched pairs signed rank test in the case of matched pairs. Indeed, since control subjects naturally lack tumors, we compared tumor amplitudes in patients against the corresponding whole-brain amplitudes in patients, denoting the corresponding p-values with a delta (Δ) symbol. These data are also presented in **Figure 3**.

**Supplemantary Table 2. Significant Spearman correlation coefficients (and the respective p-values in brackets) between region-specific MREG_BOLD_ amplitudes and clinical factors.**

| **Amplitude** | **MSKCC** | **Age** | **FLAIR** | **Maximum Gd+** | **Total Gd+** | **Multifocality** |
| --- | --- | --- | --- | --- | --- | --- |
| **Whole brain** |  |  |  |  |  |  |
| **Full Band** |  |  |  |  |  |  |
| **VLF** |  |  |  |  |  |  |
| **Respiratory** |  |  |  |  |  |  |
| **Cardiac** |  |  |  |  |  | 0.42 (0.021) |
| **Lateral Ventricle** |  |  |  |  |  |  |
| **Full Band** |  |  |  |  | 0.37 (0.047) | 0.48 (0.007) |
| **VLF** |  |  |  |  |  |  |
| **Respiratory** |  |  |  |  |  | 0.41 (0.025) |
| **Cardiac** |  |  |  |  |  | 0.43 (0.017) |
| **White Matter** |  |  |  |  |  |  |
| **Full Band** |  |  |  |  |  |  |
| **VLF** |  |  |  |  |  |  |
| **Respiratory** |  |  |  |  |  |  |
| **Cardiac** |  | 0.42 (0.022) |  |  |  | 0.39 (0.032) |
| **Grey Matter** |  |  |  |  |  |  |
| **Full Band** |  |  |  |  |  |  |
| **VLF** |  |  |  |  |  |  |
| **Respiratory** |  |  |  |  |  |  |
| **Cardiac** |  |  |  |  |  | 0.42 (0.023) |
| **Tumor** |  |  |  |  |  |  |
| **Full Band** |  |  |  |  |  | 0.43 (0.018) |
| **VLF** |  |  |  |  |  |  |
| **Respiratory** |  |  |  |  |  |  |
| **Cardiac** |  |  |  |  | 0.39 (0.034) | 0.59 (0.001) |
| **Peritumoral** |  |  |  |  |  |  |
| **Full Band** |  |  |  |  |  |  |
| **VLF** |  |  |  |  |  |  |
| **Respiratory** |  |  |  |  |  |  |
| **Cardiac** |  | 0.37 (0.047) |  |  |  | 0.39 (0.033) |

Note: ROI-specific MREG_BOLD_ amplitudes correspond to the following physiological frequency bands: full band (fB; 0.008 – 5 Hz), very-low frequency (VLF; 0.008 - 0.1 Hz), respiratory (RESP; 0.1–0.5 Hz), and cardiac (CARD; 0.7–2 Hz). MSKCC = Memorial Sloan Kettering Cancer Center scores. FLAIR = volume of FLAIR hyperintensity. Maximum Gd+ = *maximum* contrast-enhancing tumor diameter. Total Gd+ = *total* sum of all contrast-enhancing tumor diameters. Multifocality = number of contrast-enhancing foci.
